# Supplementary material for: Examination of the Virome of Taro Plants Affected by a Lethal Disease, the Alomae-Bobone Virus Complex, in Papua New Guinea
Source: Viruses. 2022 Jun 28;14(7):1410. doi: 10.3390/v14071410 (PMC9320088; doi:10.3390/v14071410)
Supplement: Supplementary file 1 [file viruses-14-01410-s001.zip › Figures S1 and S2.pdf]

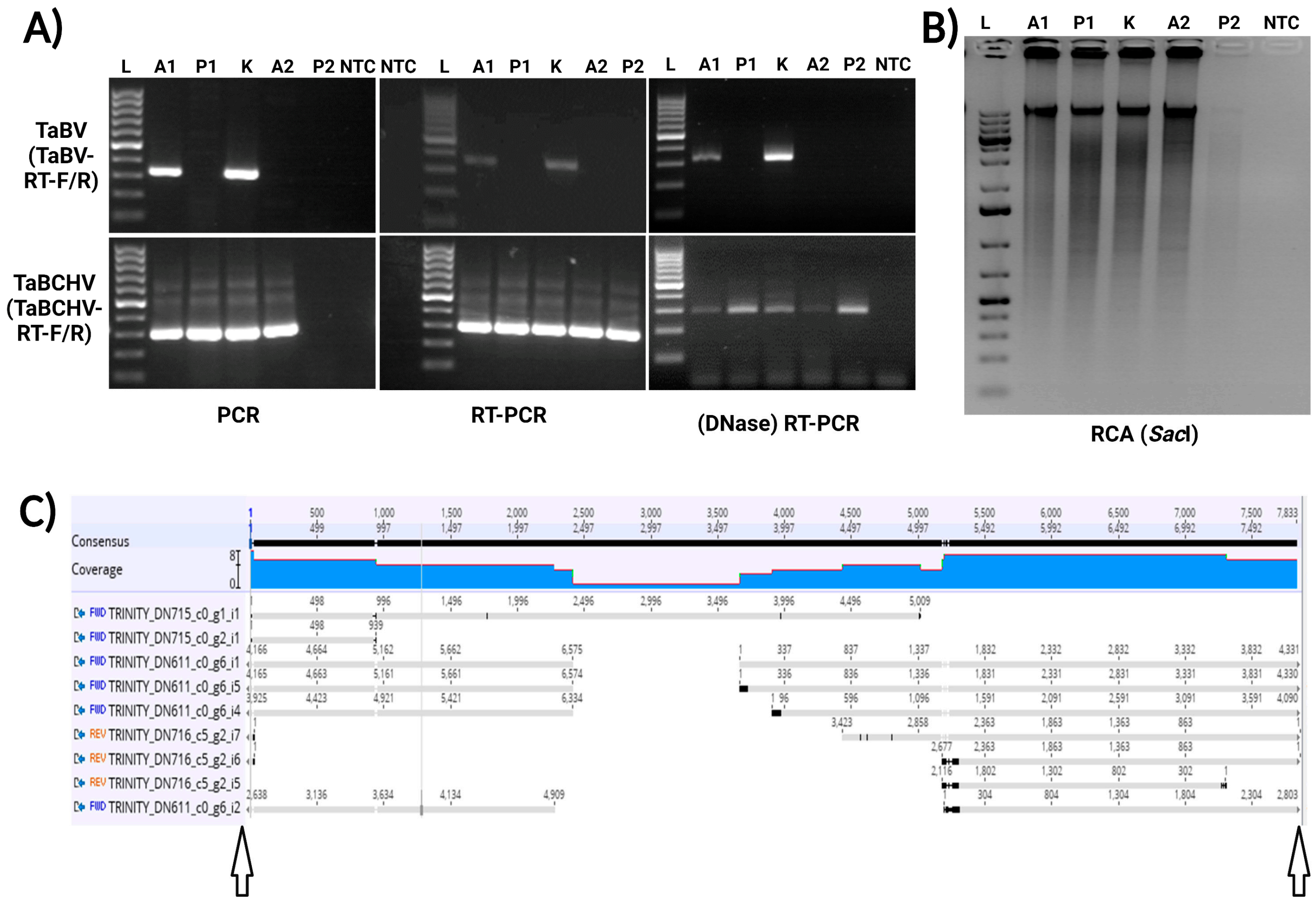

Figure S1. In vitro and in silico assays that demonstrate episomal badnavirus, TaBV and TaBCHV, presence in the taro samples collected from Papua New Guinea (PNG). L = ladder. A1 = asymptomatic sample 1 from Girua. A2 = asymptomatic sample 2 from Girua. P1 = ABVC-symptomatic sample 1 from Popondetta. P2 = ABVC-symptomatic sample 2 from Popondetta. K = ABVC-symptomatic sample from Kokoda. (A) Comparison of the results obtained on PCR, RT-PCR, and DNase-RT-PCR assays using TaBV- and TaBChV-specific primers. (B) *Sac*I digestion of RCA amplicons from the five PNG samples. (C) Mapping representation of the contigs used to obtain a circular scaffold sequence of TaBV by an iterative mapping approach. Arrows point to the continuity of the contigs beyond the linearized circular sequence of TaBV.

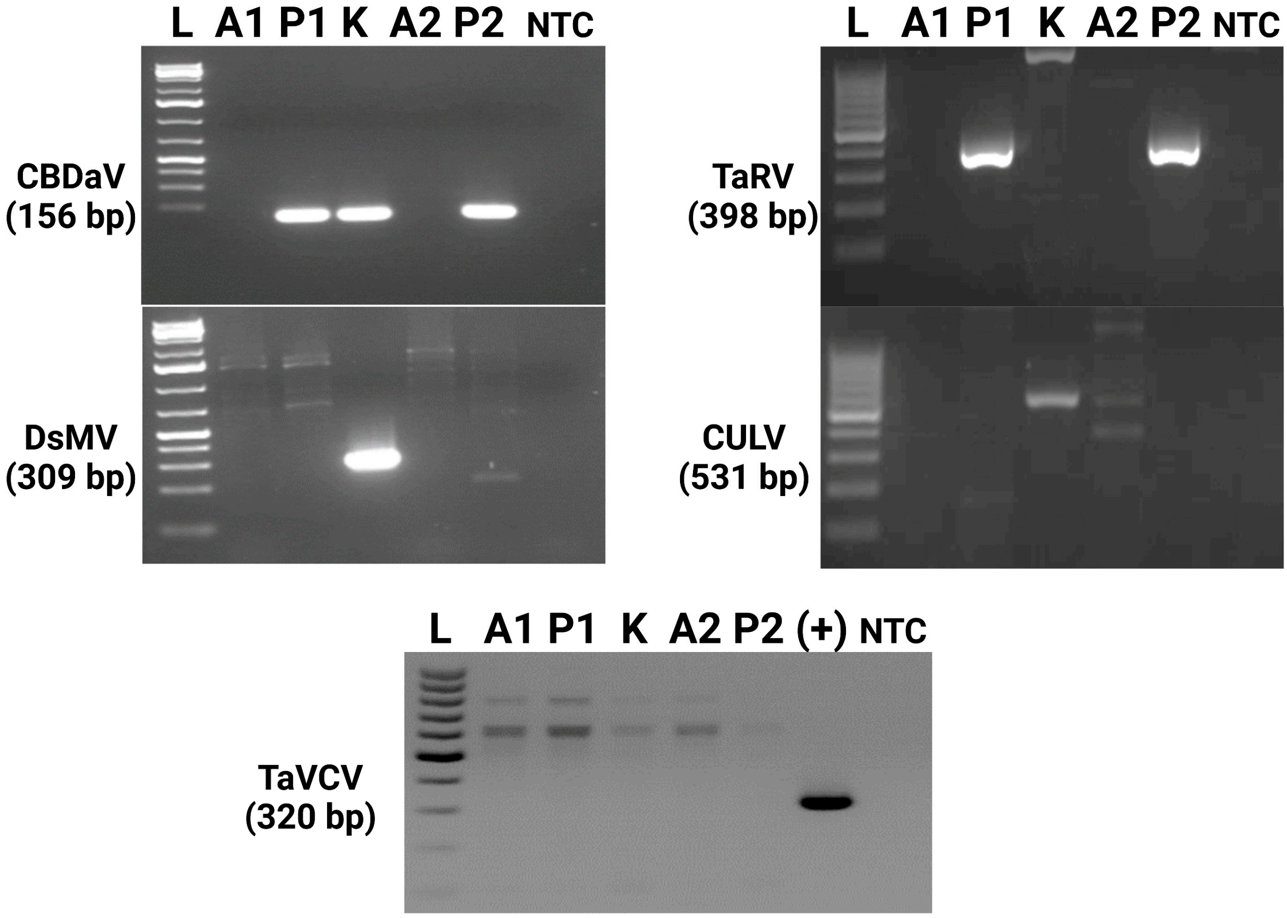

Figure S2. Agarose gel electrophoresis of the RT-PCR products used to determine virus presence in the ABVC-symptomatic samples (K, P1 and P2) and samples not presenting ABVC-related symptoms (A1 and A2). Amplicons were amplified using the virus-specific primers detailed in Table S1. Amplicons of RT-PCR assays for TaBV and TaBCHV are present in Figure S1. L = ladder. A1 = asymptomatic sample 1 from Girua. A2 = asymptomatic sample 2 from Girua. P1 = ABVC-symptomatic sample 1 from Popondetta. P2 = ABVC-symptomatic sample 2 from Popondetta. K = ABVC-symptomatic sample from Kokoda.
